# Dynamics of liquid-liquid phase separation of wheat gliadins

**DOI:** 10.1038/s41598-018-32278-5

**Published:** 2018-09-27

**Authors:** Adeline Boire, Christian Sanchez, Marie-Hélène Morel, Minne Paul Lettinga, Paul Menut

**Affiliations:** 1 UMR IATE, Université de Montpellier, Montpellier SupAgro, INRA, CIRAD, 2, Place Viala, 34060 Montpellier Cedex 1, France; 2 INRA, UR1268 Biopolymers Interactions Assemblies, 44300 Nantes, France; 3 UMR IATE, INRA, Université de Montpellier, Montpellier SupAgro, CIRAD, 2, Place Viala, 34060 Montpellier Cedex 1, France; 4 Soft Condensed Matter Group ICS3, Jülich Forschungscentrum, Jülich, Germany; 5 Department of Physics and Astronomy, Laboratory for Soft Matter and Biophysics, KU Leuven, Celestijnenlaan 200D, B-3001 Leuven, Belgium; 6 Ingénierie Procédés Aliments, AgroParisTech, INRA, Université Paris-Saclay, 91300 Massy, France

## Abstract

During wheat seeds development, storage proteins are synthetized and subsequently form dense protein phases, also called Protein Bodies (PBs). The mechanisms of PBs formation and the supramolecular assembly of storage proteins in PBs remain unclear. In particular, there is an apparent contradiction between the low solubility in water of storage proteins and their high local dynamics in dense PBs. Here, we probe the interplay between short-range attraction and long-range repulsion of a wheat gliadin isolate by investigating the dynamics of liquid-liquid phase separation after temperature quench. We do so using time-resolved small angle light scattering, phase contrast microscopy and rheology. We show that gliadins undergo liquid-liquid phase separation through Nucleation and Growth or Spinodal Decomposition depending on the quench depth. They assemble into dense phases but remain in a liquid-like state over an extended range of temperatures and concentrations. The analysis of phase separation kinetics reveals that the attraction strength of gliadins is in the same order of magnitude as other proteins. We discuss the respective role of competing interactions, protein intrinsic disorder, hydration and polydispersity in promoting local dynamics and providing this liquid-like behavior despite attractive forces.

## Introduction

Most plant seeds have the unique ability to naturally store large reservoirs of proteins in a stable and compact environment for extended periods. Specific organelles are dedicated to this storage: plant protein bodies (PBs). These PBs are current targets for producing recombinant proteins of medical interest as they provide high expression level and a protective environment^1–3^. However, the mechanisms underlying the supramolecular assembly of storage proteins into PBs are far from being understood whereas it is of both scientific and technological interest.

In wheat seeds, PBs form dense spherical accretions of about 0.5 to 2 μm in the lumen of endoplasmic reticulum (ER) once protein accumulation starts^4,5^. Wheat storage proteins were first thought to spontaneously precipitate into insoluble deposits due to their low extractability in aqueous solutions^5^. It was later shown in Xenopus oocytes that ER-deposited storage proteins were able to diffuse within the cisterna of ER demonstrating their solubility and monomeric state^6,7^. Also, PBs have been shown to be highly dynamic structures which undergo fusion/coalescence with other PBs^4,8^ and exchange their content over time^9,10^. These data suggest that PBs are dynamic bodies rather than dead bodies where proteins undergo irreversible aggregation. They show surprising similarities with membraneless organelles, highlighted in living cells like P granules^11,12^. Despite intense research effort, the contribution of the intrinsic properties of wheat storage proteins to this phenomenon has not yet been elucidated. In particular, there is an apparent contradiction between their very low solubility in water suggesting strong attractive properties and their high local dynamics in dense PBs, suggesting a peculiar equilibrium of protein-protein and protein-solvent interactions.

To tackle this question, we use an *in vitro* approach probing the phase behaviour of a wheat storage proteins isolate to infer their interaction properties as done for lysozyme^13–15^, lens proteins^16–18^ or casein micelles^19,20^. One of the challenges is to isolate a protein system close to what is found in the seed at the time of PBs formation. Two types of storage proteins are synthetized and accumulated: gliadins and glutenin sub-units. They share a similar amino acid composition as well as a similar polypeptide structure including a central domain of low complexity rich in proline and glutamine^21^. They mainly differ in their propensity to form intermolecular disulphide bonds. Gliadins remain monomeric units whereas glutenin subunits progressively assemble into polymers by oxidation of their free thiol functions: these polymers reach several millions of Da. This polymerization occurs in the late stage of seed development^22^. In the early stage of protein accumulation, PBs include a polypeptide mixture made of monomeric gliadins and glutenin subunits distributed in PBs^4^. As a first approach, we choose to work on monomeric gliadins extractible from mature seeds as a representative protein mixture at the time of PBs formation. Another challenge is to choose the appropriate solvent to probe the interaction properties of wheat storage properties. The most biologically relevant solvent to probe the interaction properties of wheat storage proteins is an aqueous solution at pH 7.2–7.5 to be close to ER conditions^23,24^. However, proteins from mature wheat seeds are poorly soluble in these conditions, which preclude the study of their interactions from diluted to condensed state in this solvent. Our approach consists in working in good solvent conditions, in 55% v/v ethanol/water mixture, and decreasing solvent quality by decreasing temperature. In a previous work, we showed that gliadins undergo a liquid-liquid phase separation upon decreasing temperature^25^. The occurrence of such liquid–liquid phase separation has been attributed to short-range protein–protein attraction^26^. In good solvent quality, we showed that gliadin dispersions remain in a liquid-like state at 20 °C for concentrations as high as 450 g/L revealing a net repulsion in the condensed system^27^. In the present paper, we study the dynamics of phase separation upon decreasing solvent conditions to probe the interplay between short-range attraction and global repulsion observed in good solvent conditions. We aim at identifying the mechanisms of phase separation and at probing the physical state of gliadin dispersions depending on temperature and concentration (T,c). Through this work, we show that gliadins are prompted to assemble into dense protein phases but remain in a dynamic state even when condensed. We show that gliadin dispersions are in a liquid-like state over an extended range of concentration (from 10 to 500 g L^−1^) and temperatures (2 to 20 °C). We provide a detailed analysis of the kinetics of phase separation from which an apparent second virial coefficient is inferred. We show that attractive properties of gliadins are of the same order of other globular proteins that would gel *via* arrested spinodal decomposition. We discuss how competing interactions, protein flexibility and the polydispersity of our system may prevent percolation by providing high local dynamics. We discuss the biological significance of these results in the framework of PBs formation.

## Materials and Methods

### Gliadin isolation

Grains from a 2–12 type wheat (cv. Haussman, 2010 harvest) were milled on a Bühler laboratory mill (MLU 202, Bühler, Switzerland) according to an approved method of the American Association of Cereal Chemists (method 26–31.01, 2000). We developed a gentle procedure to extract wheat storage proteins based on the differential solvent solubility of wheat flour proteins as described previously^27^. From this procedure, we obtained a protein powder composed mainly of gliadins (86.6% +/− 1.7) with a residual content of glutenins (7.1% +/− 1.6) and of metabolic proteins (6.4% +/− 0.8).

The protein powder was dissolved into ethanol/water solution 55% v/v, maximum solubility condition of these proteins, using magnetic stirrer to reach 10 g L^−1^. Residual undissolved solids were removed by centrifugation for 10 min at 20000 g. The dispersions were then filtered on a 0.22 μm Nylon filter (Magna), before concentration by dialysis against a 10% (wt/v) polyethylene glycol 20000 (Sigma Aldrich) dissolved into 55% v/v ethanol/water solution, NaCl 0.5 mM. Dialysis was conducted in tubes with a cut-off of 12 kDa (Spectrapore), in opaque containers at 20 °C until the protein concentration reaches 250 g L^−1^. Protein concentrations were determined by UV absorption spectroscopy. We determined a specific absorbance coefficient (A_280 nm_) of 0.570 L g^−1^ cm^−1^. The concentration c (in g L^−1^) was converted into volume fraction *Φ* using a partial specific volume *v*_*s*_ of 0.76 mL mg^−1^ ^28^.

### Determination of protein composition (SE-HPLC, Acid-PAGE)

The composition of wheat proteins was assessed by Size-Exclusion-HPLC (SE-HPLC) according to an established method^29^. The SE-HPLC apparatus (Waters model Alliance) was controlled by the Millenium software (Waters) and equipped with a TSK G4000-SWXL (TosoHaas) size exclusion analytical column (7.5 × 300 mm) and a TSK G3000-SW (TosoHaas) guard column (7.5 × 75 mm). The columns were eluted at 20 °C (+/−2 °C) with 0.1 M sodium phosphate buffer (pH 6.9) containing 0.1% SDS. The flow rate was 0.7 mL min^−1^. Protein absorbance was recorded at 214 nm. Peak areas were corrected from the SDS-phosphate buffer contribution. The elution time of every profile was converted into MW with protein standards of known MW.

Electrophoresis in Acetic Acid-Urea polyacrylamide gel (Acid-PAGE) separates proteins according to their molecular size and their charge^30^. Polyacrylamide gels (12% acrylamide, 0.375% bis-acrylamide) contained 2 M urea, 0.1% ascorbic acid, 0.0014% ferrous sulphate, and 0.75% glacial acid acetic, pH 3.1. 40 mL of the gel solution was degas for 5 min under vacuum at ambient temperature, and then 55 μL of 0.6% (v/v) H_2_O_2_ catalyst was added to cast one gel (160 × 180 × 0.75 mm). Electrophoresis was performed for 3 hr 45 min at 500 V at 18 °C. After electrophoresis, the gels were incubated in 15% trichloroacetic acid overnight, rinced with tap water for 5 min and stained in 12.5% trichloroacetic acid with 0.14% (w/v) Coomassie Brillant Blue R250. After staining, both gels were rinced with tap water and pictured.

### Small-Angle X-Ray Scattering (SAXS)

SAXS experiments were performed on the SWING beamline at Synchrotron SOLEIL, Gif-sur-Yvette, France. The sample-to-detector distance was fixed to 1.014 m and X-ray energy was 12.0 keV. The exploitable q-range was 10^−2^ − 1 Å^−1^, where *q* = 4*π* sin *θ*/*λ*, with 2*θ* the scattering angle and *λ* the wavelength. The samples were loaded in a thermostated kapton capillary. A total of 15 frames of 2 s each were recorded. In all cases, the transmitted intensity was measured using a diode embedded in the beam-stop. The recorded curves were normalized to transmitted intensity and subsequently averaged using Foxtrot software. The same protocol was applied to buffer scattering. Form factor fitting was performed using SASVIEW software.

### Dynamic Light Scattering (DLS) and Electrophoretic mobility measurements

DLS and electrophoretic mobility measurements were performed with a Malvern Zetasizer Nano ZS (Malvern, Herrenberg, Germany) equipped with a 633-nm He-Ne laser and operating at an angle of 173°. For DLS, 500 μl of each sample, filtred on 0.45 μm and 0.2 μm filters, was measured in single-use polystyrene half-micro cuvettes (Fisher Emergo, Landsmeer, The Netherlands) with a pathlength of 10 mm. Measurement position in the cuvette was automatically fixed as well as the attenuator position. Data were collected in manual mode: 10 runs of 30 seconds were collected and repeated three times for each measurement. The error bars displayed were obtained by the standard deviation (SD) of three samplings of the same sample. The intensity size distributions were obtained from the autocorrelation function using the Contin mode. For electrophoretic mobility, 500 μl of each sample, filtred on 0.45 μm and 0.2 μm filters, was measured in Folded Capillary Zeta Cell DTS1070. Data were collected in automatic mode.

### Time-resolved small-angle light scattering (TR-SALS)

A home-made high-pressure temperature-controlled cell was used for all experiments to hold the samples. The sample holder consisted of a brass ring which has on its periphery a number of small holes closed by two sapphire windows and a Viton O-ring. A surrounding jacket in which water circulates was used to control the temperature of the sample. The temperature accuracy was within 1 K. A nitrogen flow was used to avoid water condensation on the sample holder during cooling. A 15 mW HeNe laser (Melles Griot) operating at a wavelength of 632.8 nm was directed through the center of the sapphire windows of the cell^31,32^.

Scattered light was directly projected with a lens on to the chip of a Peltier-cooled 12-bit CCD camera with 582782 pixels (Princeton Instruments, microMAX). A strip of a neutral density filter was used as beam-stop so that the scattered intensity could be corrected for the turbidity by division with the residual intensity transmitted through the beam stop. The scattering angles on the chip were calibrated by placing a known grid PAT 13 Heptagon at the sample position. The time evolution of scattered light intensities was monitored at scattering angles from 2.6 to 13.6°. The sample was loaded in the pre-cooled sample holder in about 10 seconds. The recording was started before sample loading, time 0 was chosen once the sample was on place.

### Light microscopy

The microscope Olympus BX53 was equipped with a Linkam PE60 Peltier stage. The objective Plan 40x with a numerical aperture of 0.5 was used. Images were recorded on an Olympus DP26 digital camera with a time frame of 15 frames per second. The recording of images was started at 20 °C after which temperature quenches were performed.

The images were Fourier transformed using ImageJ, giving radial averaged intensity profiles as function of the distance,*d*, from the center of the spectrum. The distance *d*, in pixel, can be converted in a characteristic wavelength *L*(*t*) using Eq. 1^33^:

1 $${\boldsymbol{L}}({\boldsymbol{t}})={\boldsymbol{w}}\times {\boldsymbol{s}}/{\boldsymbol{d}}$$

where *w* is the width in pixel of the image analyzed and *s* the scale ratio (nm/pixel). The wave number *q*^*FTT*^ in nm can be calculated using the Bragg’s formula reported in Eq. 2:

2 $${q}^{FTT}=2\pi /{\boldsymbol{L}}({\boldsymbol{t}})$$

### Rheological measurements

The rheological properties of wheat gliadins dispersions were investigated for *Φ* > 0.05 using a stress-controlled AR 2000ex rheometer (TA Instruments, USA) equipped with a 20 mm and 4° cone plate geometry. We used a solvent trap and saturate the environment with the solvent vapor to prevent evaporation. Dynamical measurements, at 0.1 Hz, 3% of strain, were performed upon temperature decrease from 20 to 2 °C with a temperature ramp of 20 K min^−1^ using a Peltier plate. By the end of the dynamical measurement, the sample was at equilibrium, *i*.*e*. constant G′ and G″ over time. A frequency sweep was then performed over a frequency range of 0.05–60 Hz at 3% of strain at 2 °C. A final dynamical measurement at 0.1 Hz, 3% of strain and 20 °C was performed during 10 minutes to check that the sample recover its initial rheological properties.

## Results

### *in vitro* model for wheat prolamins

**Figure 1:**
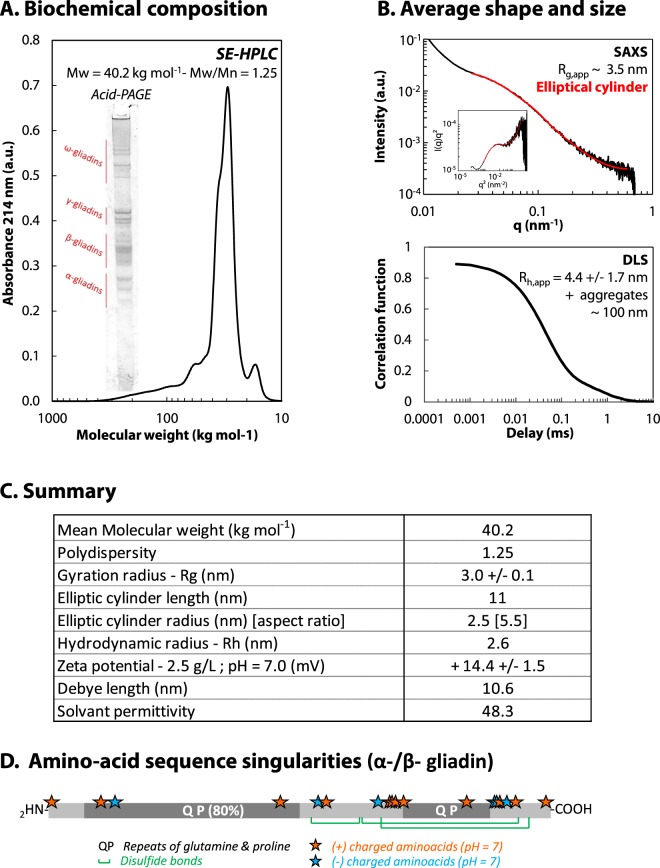
Biochemical and structural characterization of the gliadin isolate. (**A**) Size-exclusion profile of the isolate eluted in 0.1 M sodium phosphate buffer, pH 6.9, containing 0.1% SDS at 20 °C. The MW range effectively separated by the column is comprised between 10 and 1000 kg mol^−1^. Inset: Acid-PAGE electrophoresis. (**B**) Scattering intensity obtained by SAXS at 25 g L^−1^ in 55% v/v water−ethanol mixture, 0.5 mM NaCl at 20 °C. Inset: Kratky plot. Solid red lines stand for the form factor of an elliptic cylinder. Correlogram obtained by DLS at 25 g L^−1^ in 55% v/v water−ethanol mixture, 0.5 mM NaCl at 20 °C. (**C**) Table summarizing of gliadin isolate’s main characteristics. (**D**) Scheme of α-/β- gliadin amino acid sequence (UniProtKB - P18573) with charged amino acids (stars), glutamine and proline repeats (QP) and disulfide bonds (green).

We developed a gentle extraction procedure to isolate wheat gliadins. The isolate is composed by a blend of proteins with a mean molecular weight (MW) of 40.2 kg mol^−1^ and a polydispersity index of 1.25 as shown by Size-Exclusion chromatography (SE-HPLC) performed in presence of an anionic detergent in order to disrupt all weak interactions between proteins (Fig. 1A). Gliadins (25 < MW < 70 kg mol^−1^) represent 86.7 ± 1.7% of the isolate. Several isoforms of each gliadin type are present in the mixture as shown by Acid-PAGE in the inset of Fig. 1A even though we worked with flour from a single wheat cultivar. It is part of the intrinsic diversity of wheat proteins.

We characterized “apparent” structural features of gliadin dispersions by considering gliadins as a whole. The structure of wheat gliadin dispersions was investigated using small-angle X-ray scattering (SAXS). Scattered intensity (*I*) obtained at 25 g L^−1^ is plotted as a function of the scattering vector (*q*) in Fig. 1B. The upturn of the scattering intensity at small *q* is ascribed to aggregation, but might also result from forward scattering^34^. For *q* > 0.02 nm^−1^, *I*(*q*) can be fitted by a form factor of an elliptic cylinder. We find an average length of 11 nm, a major radius of 2.7 nm and an aspect ratio of 5.5 giving an apparent gyration radius (*R*_*g*,*app*_) of 3.5 nm. These dimensions are in good agreement with results already published in the literature^35–38^. Each type of gliadins has been successfully modelled by a rod model in a dilute dispersion in 70% v/v ethanol−water mixture. They share a similar diameter but differ by their length and therefore by their aspect ratio^39^. We used Dynamic Light Scattering (DLS) to determine an apparent hydrodynamic radius (*R*_*h*,*app*_). The autocorrelation function, displayed in Fig. 1B, suggests two populations of scatters in the protein dispersion at 25 g L^−1^: a major population with *R*_*h*,*app*_ = 4.4 +/−1.7 nm and a minor population with *R*_*h*_ = 108 +/−28 nm corresponding to protein clusters. The size of these clusters does not depend on protein concentration nor on temperature for *c* < 30 g L^−1^ and *T *> 12.5 °C respectively (data presented in Supplementary data 1). Their size is also insensitive to ionic strength up to 0.5 M, but their volume occupancy decreases with increasing ionic strength (data not shown). This behavior ressembles to mesoscopic clusters observed in other protein dispersions such as lysozyme^40,41^ and glucose isomerase^41^. It has been suggested that mesoscopic clusters do not affect the bulk solution because of their low volume fraction, estimated between 10^−7^ and 10^−3^ ^40,42^. The presence of such clusters in gliadin dispersions deserves further investigation but is beyond the scope of the present paper.

We also probed the electrostatic potential of gliadin dispersions using electrophoretic mobility measurements, which provide information on the effective charge of the molecules surrounded by ions in solution. A mean zeta potential of 14.4 +/− 1.5 mV was obtained at 2.5 g L^−1^. Using DLVO theory, this gives a relatively small electrostatic potential below 0.5 *k*_*B*_*T* suggesting that there is a weak electrostatic repulsion between proteins.

### Gliadins display two mechanisms of phase separation

We investigated the kinetics of phase separation using TR-SALS to identify the mechanism of phase separation depending on the (T,c) conditions. Two mechanisms of phase separation can occur depending on the position of the quench within the phase diagram^43^. Nucleation and growth (NG) occurs if the system is quenched into the metastable region with ∂Π/∂*ρ* > 0 between the binodal and the spinodal. In this case, phase separation will be initiated only if localized, large amplitude concentration fluctuations associated with the formation of a nucleus occur. Droplets with equilibrium composition will then grow. The probability of forming a nucleus with a concentration significantly different from the surroundings being small, this leads to the existence of an induction time. Spinodal decomposition (SD) occurs if the system is quenched below the spinodal line. The system will be unstable and mass transfer against concentration gradient are favoured, so that ∂Π/∂*ρ* < 0. Homogeneous concentration fluctuation will grow instantaneously at one characteristic length. Once the fluctuations reach the equilibrium composition, the size of the fluctuations will grow in size. Arrested spinodal decomposition may occur if the continuous protein-rich phase reaches glass transition leading to a non-equilibrium state and a solid-like network^13,14,44^. The time dependence and space resolution of the concentration fluctuations are governed by the mechanism involved and depend on diffusive and interaction properties in the system^43^.

**Figure 2:**
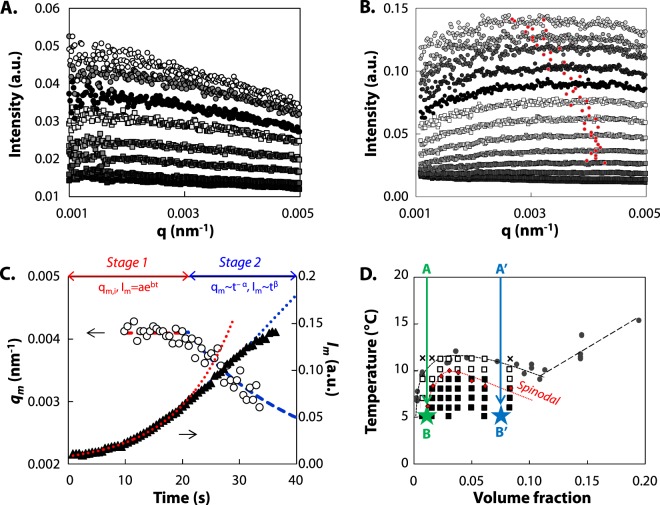
Dynamics of phase separation of gliadins dispersions investigated by TR-SALS. (**A**) Time dependence of the scattered light intensity versus scattering vector, *I*(*q*), for a protein dispersion of *c*= 30 g L^−1^ quenched from 20 °C to 10 °C. The time increment is 90 seconds. (**B**) Time dependence of *I*(*q*) for *c*= 30 g L^−1^ and from 20 to 6 °C. The time increment is 2.25 seconds; red circle stands for the intensity maximum found by fitting the scattering data. (**C**) Time dependence of the peak position *q*_*m*_ (empty circle) and the peak height *I*_*m*_ (solid triangle) for *c* = 30 g L^−1^ from 20 °C to 6 °C. During stage 1, *q*_*m*_ remains constant and *I*_*m*_ follows an exponential law (red dashed and dotted lines). During stage 2, *q*_*m*_ and *I*_*m*_ follow a power law with time (blue dashed and dotted lines). (**D**) Phase diagram of gliadins. Circles: experimental cloud point curve determined in^25^, Empty squares: pattern 1 in TR-SALS, Black filled squares: pattern 2 in TR-SALS, Crosses: no phase separation in TR-SALS, Red filled diamonds: spinodal temperatures determined from *I*(*q*, *t*), during spinodal decomposition (see text for details). Dashed and dotted lines are guide to eye for the binodal (black) and spinodal (red). Stars and associated letters indicate the phase contrast microscopy conditions presented in Fig. 4A.

We performed thermal quenches on protein dispersions and monitored over time the intensity of scattered light at different scattering angles. The temporal evolution of the scattering function as a function of scattering vector, *I*(*q*) *vs q*, is indicative of the mechanism of phase separation. Two types of scattering pattern were observed, depending on the protein concentration *c* and on quench temperature *T*. These two patterns are illustrated in Fig. 2A,B for *c* = 30 g L^−1^ at *T* = 10 °C and 6 °C, respectively. Pattern 1 displays a monotonous decrease of the scattering intensity as a function of *q*, indicative of NG (Fig. 2A). Pattern 2 displays a maximum in intensity that develops rapidly at a nonzero scattering vector (*q*_*m*_) (Fig. 2B). A ring in scattering pattern, though commonly attributed to SD, can also be observed in NG. In such a case, it could be observed either at low particles concentrations, when a depletion layer surrounds the particles builds up, or at high concentrations due to the positioning of individual scatters in a constrained space^45^. As the peak evolution with time strongly differs depending on the phase separation mechanism, one can distinguish both processes based on the kinetics. Two main stages in the peak evolution were found for pattern 2 as reported in Fig. 2C. During the first stage, the peak position remains constant while the intensity follows an exponential growth, followed by a shift towards smaller q vector with time (stage 2). Both, the temporal evolution of the peak position and the initial exponential increase of *I*_*m*_ at fixed *q*_*m*_ are indicative of SD^46–51^. The locations of scattering patterns 1 and 2 are plotted with the already established cloud point curve in Fig. 2D^25^, showing a good agreement between the cloud point temperatures and the emergence of NG (empty squares). The location of the spinodal can be inferred from the boundary between the pattern 1 region (empty squares) and pattern 2 region (solid squares). The phase diagram is plotted as a function of volume fraction for comparison with colloidal and protein phase diagrams.

**Figure 3:**
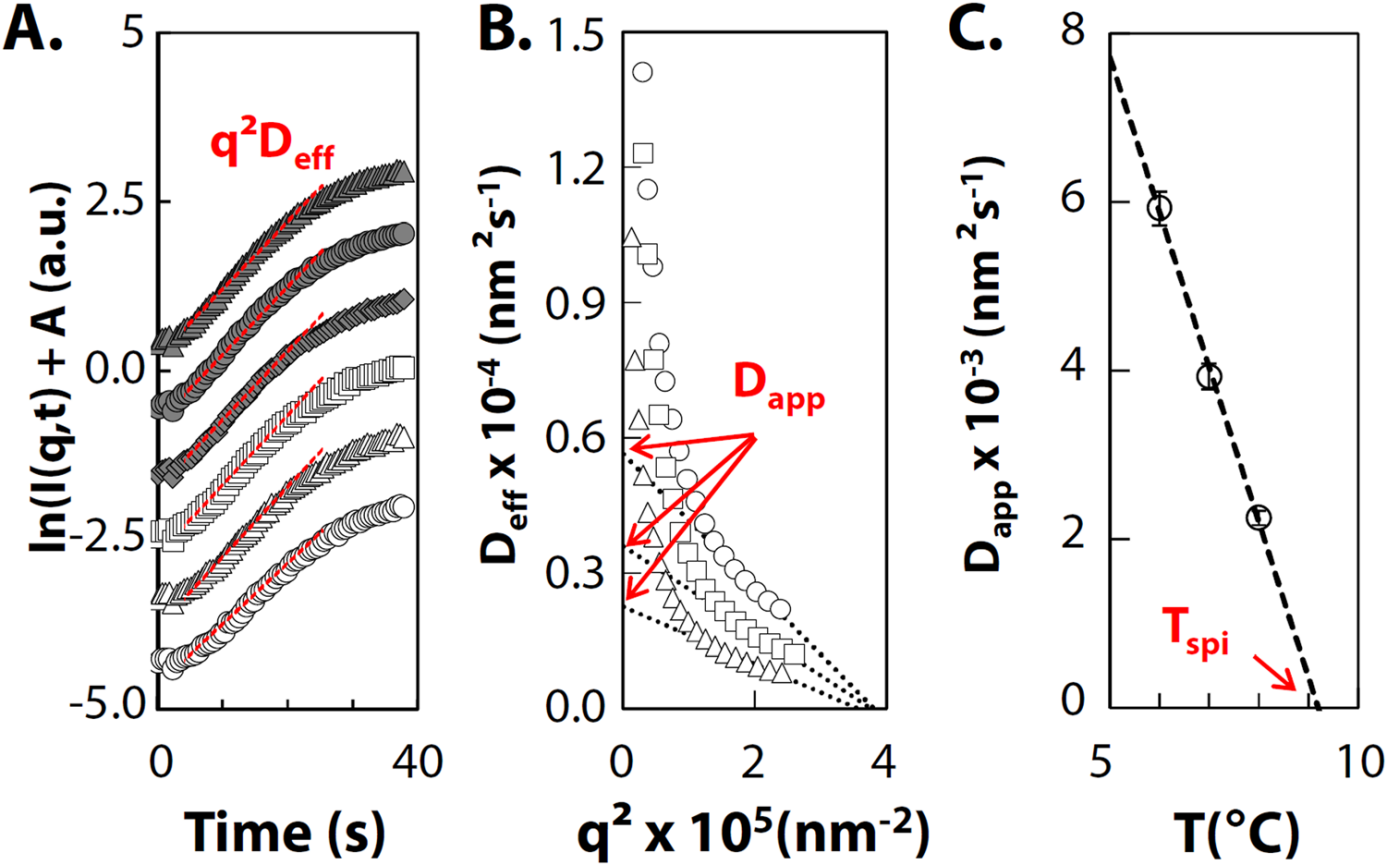
Determination of spinodal temperatures using the kinetics of phase separation. (**A**) Scattered intensity as a function of time shifted, for clarity, by a factor A for *q* = 0.002, 0.003, 0.0035, 0.004, 0.0045 and 0.005 from the bottom to the top for *c* = 30 g L^−1^ from 20 °C to 6 °C. (**B**) The growth rate of the fluctuation *D*_*eff*_ vs *q*^2^ observed at 6 °C (circle), 7 °C (square) and 8 °C (triangle) during stage 1, for *c*= 30 g L^−1^. The apparent diffusion *D*_*app*_ and the maximum wavenumber *q*_*m*_ are determined for 2.0 10^−3^ < q < 3.5 10^−3^ nm^−1^. (**C**) *D*_*app*_ as a function of temperature for *c* = 30 g L^−1^. Scale bars represent error propagation due to extrapolation in Fig. 3B. The spinodal temperature *T*_*spi*_ is determined by extrapolation of the data to *D*_*app*_ = 0.

Spinodal temperatures *T*_*spi*_ can be more precisely determined using Cahn Hilliard theory and the formalism of Dhont^51,52^. The recorded intensity *I*(*q*,*t*) in pattern 2 can be written as *I*(*q*, *t*) ∝*exp*(−D_*eff*_*q*^2^*t*) where *D*_*eff*_ is the effective diffusion coefficient:

3 $${D}_{eff}={D}_{0}\beta (\frac{\partial \Pi }{\partial \rho }+\Sigma {q}^{2})$$

with *D*_0_ the self-diffusion of the particles, *β* = 1/*k*_*b*_*T* with *k*_*b*_ the Boltzmann constant, *T* temperature. ρ is the number density. It equals to *cN*_*A*_/*M*, with *N*_*A*_ Avogrado’s constant and M molecular weight. ∂Π/∂*ρ* is the osmotic compressibility and Σ is the square gradient term. *D*_*eff*_ → 0 at the spinodal line so that *q*_*m*_ the *q*-value at which growth is maximum is given by

4 $${q}_{m}^{2}=-\frac{\frac{\partial \Pi }{\partial \rho }}{\Sigma }$$

which is one of our observables. With that we find

5 $${D}_{eff}={D}_{app}(1-\frac{{q}^{2}}{{q}_{m}^{2}})$$

where

6 $${D}_{app}={D}_{0}\beta \frac{\partial \Pi }{\partial \rho }$$

is the apparent diffusion. ∂Π/∂*ρ* = 0 for *T* = *T*_*spi*_, and therefore *D*_*app*_ vanishes. From our experimental data, we estimate *D*_*eff*_ from the initial slope of the semi-logarithmic plot of *I*(*q*, *t*) *vs t* as illustrated in Fig. 3A. *D*_*app*_ is then determined from the intercept of *D*_*eff*_ at *q* = 0. As at small *q* the plot *D*_*eff*_ vs *q*^2^ deviates greatly from the linear relationship predicted by Cahn-Hiliard theory, we determine *D*_*app*_ by considering in Fig. 3B a restricted *q*-range from 3.5 to 5 10^−3^ nm^−1^ as done elsewhere^46,53^. Similar deviations have been observed in other systems including polymer and colloidal systems^48,54,55^ and on metal alloy and were attributed to the existence of thermal fluctuations^56^. Finally, we extrapolate the temperature dependence of *D*_*app*_ towards *D*_*app*_ = 0 as illustrated on Fig. 3C for *c* = 30 g L^−1^, where we find *T*_*spi*_ = 9.2 with an uncertainty of 0.1 °C due to extrapolation error. We extend this analysis for the whole range (*Φ*, T) investigated. Resulting *T*_*spi*_ are plotted as red diamonds in the phase diagram in Fig. 2D. The resulting shape is very similar to the cloud point curve, but shifted to lower temperatures. It separates two thermodynamic areas below the gas-liquid phase boundary: the metastable region where ∂Π/∂*ρ* > 0, between the cloud points and the spinodal line, and the unstable region where ∂Π/∂*ρ* < 0, below the spinodal line.

### Microstructures associated with the phase separation with no sign of arrested phase separation

**Figure 4:**
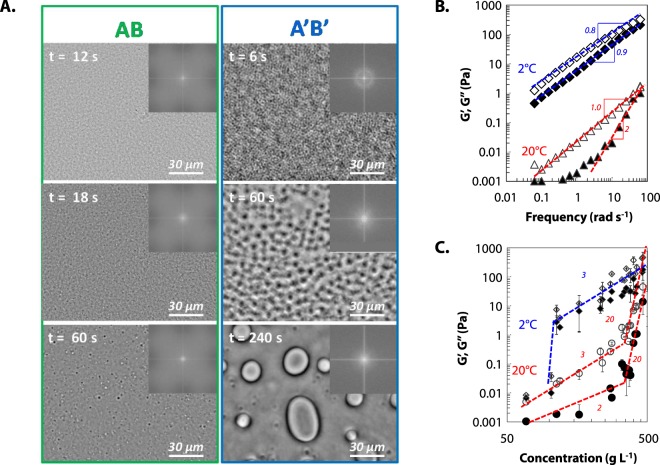
(**A**) Temporal evolution of the phase separation of protein dispersion at *c* = 10 g L^−1^ (AB) and 100 g/L (A’B’) after temperature quench from 20 °C to 5 °C observed by phase contrast microscopy. (**B**) Frequency dependence of the storage modulus G′ (filled symbols) and the loss modulus G″ (empty symbols) of gliadins dispersions at 20 °C (bottom) and 2 °C (top) for *c* = 110 g L^−1^ in 55% v/v ethanol/water solution (NaCl 0.5 mM). Power law exponents are reported. (**C**) Concentration dependence of G′ (filled symbols) and G″ (open symbols) of gliadins dispersions at 20 °C (bottom) and 2 °C (top). Power law exponents are reported.

The existence of NG and SD was confirmed using phase contrast microscopy for two thermodynamical paths reported on Fig. 2D. Paths AB and A’B’ are chosen on each side of the critical point. For each path, the temporal evolution of the microstructure is reported in Fig. 4A.

For path AB, concentration fluctuations are observed 12 s after the temperature quench as illustrated by the apparent local heterogeneity. After 18 s, a great number of droplets is visible with an apparent diameter of about 0.5–1 μm. The size of the droplets increases with time while their number decreases. On the Fourier transform spectra, we observe a monotonous decrease of the intensity. We attribute the droplet formation to the nucleation of a concentrated protein phase in a protein poor phase as *Φ* is below the critical volume fraction, *Φ*_*c*_. For path A’B’, an interconnected two-phase structure is observed with a characteristic wavelength, revealed by a ring on the Fourier transform. The characteristic wavelength corresponds to a *q-*vector of 0.005 nm^−1^, which is in the order of magnitude of the TR-SALS results.

It is noticeable that each pathway ends-up with droplet-like structure. This suggests that final states are most probably liquid phase separated states. We further confirm the absence of gelation using bulk rheology upon temperature quenches at 2 °C. A decrease in temperature induces an increase of both G′ and G″ for a protein dispersion at *c* = 110 g L^−1^, but G″ remains higher than G′ for all frequencies between 0.1 and 100 rad s^−1^ as shown on Fig. 4B. At 20 °C, the protein dispersion behaves at high frequency as a viscous liquid with G′ and G″ proportional to *ω*^2^ and *ω*, respectively, where *ω* is the frequency. At 2 °C, both exponents decrease down to 0.8 for G″ and 0.9 for G′. Thus the decrease of temperature leads to a rheological change from a viscous liquid to a visco-elastic fluid without an “arrested” phase separation as observed for lysozyme^13,14,57^ or attractive colloids^58^. The phase-separation process is therefore not interrupted by the formation of an attractive glass in the dense phase leading to an “out-of-equilibrium” gel^59^. We further checked for the presence of “equilibrium” gels, i.e. without encountering phase separation as observed for patchy colloids^59^, by quenching gliadins dispersion away from the phase boundary (Fig. 4C). No rheological signature for gelation is observed at 2 °C for protein concentration up to 500 g L^−1^. This is clearly in contrast with attractive colloids that would either form “out-of-equilibrium” gel through arrested phase separation or “equilibrium” gels.

### Attractive properties of gliadin inferred from SD kinetics

**Figure 5:**
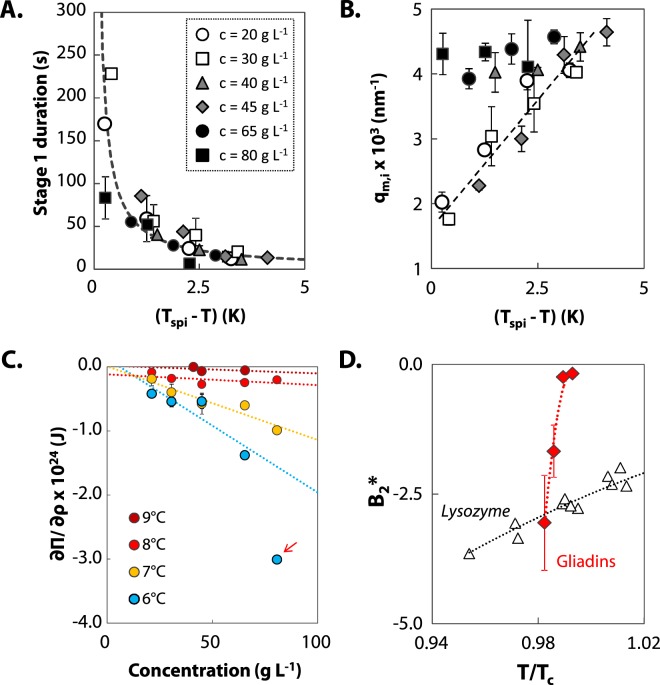
Analysis of early stage SD and interaction properties. (**A**) Duration of stage 1 of SD in gliadin dispersions as a function of quench depth (*T*_*spi*_-*T*) at *c* = 20 (empty circle), 30 (empty square), 40 (grey triangle), 45 (grey diamond), 65 (black circle) and 80 g L^−1^ (black square). The dashed line stands for an exponential best fit of the experimental data. (**B**) Quench depth dependence of the experimental initial position *q*_*m*,*i*_ (symbols as in B). The dashed line is a guide to the eye. (**C**) Osmotic compressibility as a function of protein concentration at *T* = 9 °C (dark red), 8 °C (red), 7 °C (yellow), 6 °C (blue). Dotted lines stand for a second virial expansion of the equation of state. Arrow indicates a data point that deviates from second virial expansion, thus not taken into account for the extrapolation. (**D**) Temperature dependence of the reduced second virial coefficient *B*_2_^*^ of gliadins (red diamonds) and lysozyme (triangles, data taken in the literature^14^). Dotted lines are a guide to the eye.

The absence of gelation could suggest weak attractive forces in gliadin dispersions. In this section, SD kinetics are analyzed in more details to get an insight into the interaction properties of gliadins. During the initial stage of SD, stage 1, homogeneous concentration fluctuations grow instantaneously at one characteristic length until it reaches equilibrium compositions. For gliadin dispersions, duration of stage 1 decreases exponentially with increasing quench depth as reported on Fig. 5A. Concentration fluctuations grow faster for deeper quenches as the driving force is stronger. Quench depth also affects the range of the initial concentration fluctuation as shown in Fig. 5B for *c * < 45 g L^−1^: the deeper the quench, the larger is *q*_*m*,*i*_. These results are consistent with Eqs 3 and 4, which relates the effective diffusion coefficient *D*_*eff*_ and the wavevector *q*_*m*_ of the fluctuation to the osmotic compressibility ∂Π/∂*ρ* that depends on the interaction potential of gliadins. As the quench depth increases, the attraction in the system increases, inducing faster kinetics as well as larger concentration fluctuations. For *c *> 65 g L^−1^, a weaker dependency of *q*_*m*_ to quench depth is observed. It is however not clear whether this weaker dependency is real or whether it is biased by the edge of the *q*-vector measurement window accessible with the set-up (from 1.10^−3^ to 5.10^−3^ nm^−1^).

From the scattered light intensity analysis presented in section 2, we found values for the apparent diffusivity *D*_*app*_ between 1.10^3^ and 4.10^4^ nm² s^−1^, depending on protein concentration and quench depth. These values are used to estimate ∂Π/∂*ρ* through Eq. 6. *D*_0_ was determined experimentally by DLS. We checked that the size of the two populations of scatters detected at 20 °C (Fig. 1B) is temperature independent (see Supplementary data 1). In the calculation of ∂Π/∂*ρ*, we only consider the shorter decay to evaluate *D*_0_. Indeed, during spinodal decomposition any small density fluctuation in the system will grow, so this mechanism is driven by the dominating population of non-aggregated proteins. We find that ∂Π/∂*ρ* decreases with increasing quench depth and increasing concentration as shown on Fig. 5C. The concentration dependence of osmotic compressibility is related to the interaction potential of the proteins. A second-order virial expansion of the equation of state, given by Eq. 7, relates ∂Π/∂*ρ* and the second-order virial coefficient *B*_2_ in diluted conditions:

7 $$\frac{\partial \Pi }{\partial \rho }={k}_{B}T(1+2{B}_{2}\rho )$$

This second-order virial expansion assumes two-body interactions. As we work on a mixture of proteins, we estimate an average second virial coefficient *B*_2,*ave*_ for the mixture, by extrapolation of ∂Π/∂*ρ* towards infinite dilution for each temperature. This gives an order of magnitude of the interaction properties. The experimental ∂Π/∂*ρ* deviates from the extrapolation for high protein concentration as indicated by arrow in Fig. 5C. To compare gliadins with others proteins, we calculate the reduced second virial coefficient *B*_2_^*^ = *B*_2_/*B*_2,*HS*_, with *B*_2,*HS*_ the second virial coefficient of the equivalent hard sphere. *B*_2,*HS*_ is equal to 2/3*πσ*_*eff*_^3^ with *σ*_*eff*_ the effective diameter accounting for hard core and electrostatic repulsion^60,61^. The effective radius of gliadins is about 3.3 +/−0.3 nm as determined in Supplementary data 2 based on our previous results of osmotic compressibility^27^. The resulting ${B}_{2,ave}^{\ast }$ is reported in Fig. 5D. Despite of the uncertainty in the obtained values, ${B}_{2,ave}^{\ast }$ is of the same order of magnitude of other protein systems such as lysozyme. This suggests that the absence of gelation is not related to weak attraction. Note, however, that the temperature dependence of *B*_2_^*^ of gliadins is stronger than the one observed experimentally on lysozyme^14^. Strong temperature dependence has been predicted for patchy colloids^62^. In case of a small number of strongly attractive patches, authors showed that *B*_2_^*^ remains positive over a large range of temperatures and drastically decreased upon a small additional increase of attraction.

These preliminary results should be confirmed in the future on pure gliadin dispersions with static-light scattering. Further research are needed to clarify the individual role of each gliadin type in the mixture. In a previous work, we showed that alpha-, beta- and gamma-gliadins have a very similar phase diagram suggesting similar interaction potentials in our physicochemical conditions^25^. As they account for more than 80% of the protein mixture, we believe that our results are applicable to these three types of gliadins. On the other hand, we showed that omega-gliadins phase separate at lower volume fraction than alpha-, beta- and gamma-gliadins suggesting stronger attractive properties. It remains to be tested that changing gliadin type ratios does not change the mechanical properties.

## Discussion

We showed that gliadins phase separate in dense phases following either Nucleation and Growth or Spinodal decomposition. We also showed that gliadins remain in a liquid-like state over an extended range of concentration and temperature. The system does not fulfill the two requirements for gelation: a sufficiently large relaxation time and build-up of a percolated network. Though not quantitative, one observation suggests that large relaxation times could be reached: we provided evidence for both attractive forces and repulsive barrier^27^. Combination of these conflicting forces could increase bond life time in accordance to a theoretically study showing for simple potentials that a repulsive barrier strengthens trapping of particle in the attractive well^63^. The presence/absence of a percolating network is more questionable. In principle, anisotropic shape of gliadins should lead to higher excluded volume lowering the critical volume fraction for geometric percolation compared to spherical objects^64^. However, this is not the case. Instead of percolating, gliadins at high concentration in good solvent conditions showed a low-q correlation peak in SAXS^27^, revealing a heterogeneous microstructure. The occurrence of a low-*q* correlation peak in protein dispersions has been interpreted as an intermediate range order (IRO) between scattering centers^65,66^. Such IRO is observed in conditions favoring competing interactions: short range attractive and long-range repulsive potential. Interestingly, the existence of IRO in lysozyme dispersions has been shown to be associated with a macroscopic liquid-like behavior at high volume fraction^67^. In gliadin dispersions, such IRO might corresponds to clusters as it occurs at high *Φ* and as the peak intensity increases with *Φ*^27^. The electrostatic repulsive potential of gliadins is relatively weak. However, charged amino acids are not evenly distributed along the protein but rather concentrated in the C-term domain as shown in Figure 1D. This may lead to high electrostatic repulsion depending on proteins orientation. The existence of IRO at high *Φ* may provide local glassy motion, while the voids associated with IRO would lead to diffusion at long time scale and macroscopic liquid behavior. The presence of competing interactions and the subsequent formation of IRO constitute one working hypothesis for explaining the liquid-like behavior of gliadins at high concentration. Other parameters can also play a role in this phenomenon. We showed, for example, that gliadins are prompted to change their conformation upon increasing concentration^27^. This degree of flexibility may induce compressibility and prevent percolation. The flexibility is probably provided by the N-term domain of gliadins which is predicted to be disordered. As this domain is also highly hydrophilic, a competition between protein-protein and protein-solvent interactions may also play a role in preventing percolation. Yet another parameter could interfere with percolation: the polydispersity of our protein mixture which is intrinsic of wheat storage proteins. Using numerical simulation, it was shown that polydispersity smears out the glass transition in relation with a decoupling of the dynamics of the smallest and largest particles^68^. These hypotheses need to be explored in future work. From a biological perspective, this liquid-like state at high concentration displays several advantages as compared to crystal and/or jammed phase: it provides a close packing of biomolecules while maintaining high local dynamics and favoring high diffusion^69^. Liquid-like states provide dense phases that can deform, flow and be easily mobilized. It may be a strategy to store as many amino-acids as possible while being easily dispensable if needed. Liquid-like behavior of plant PBs deserves now to be demonstrated *in vivo*. Good indications for such behavior are high exchange rate observed between PBs^9,10^ and PBs fusion/coalescence^4,8^. The development of promising techniques such as Brillouin Microscopy^70^ combined with Refractive Index Tomography^71^ could help in probing the physical state of PBs in the seed.

**Figure 6:**
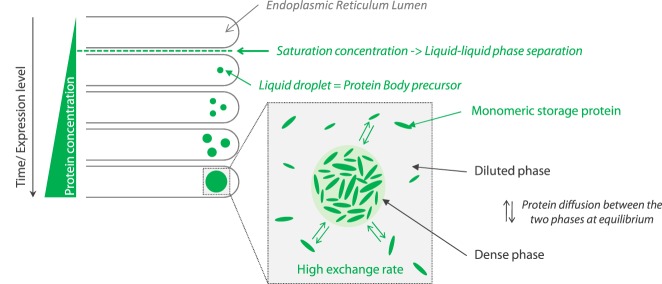
Role of liquid-liquid phase separation in the initiation of wheat PBs. Wheat storage proteins are synthetized as monomeric species in the lumen of the endoplasmic reticulum. Overtime, their local concentration increases reaching a saturation concentration over which liquid-liquid phase separation occurs leading to dense liquid droplets. Liquid droplets may grow by diffusion of monomeric proteins and/or by coalescence of several droplets. Droplets are in equilibrium with the continuous diluted phase leading to a dynamic exchange of the proteins between the two phases. We speculate that such dense liquid droplets act as precursor for wheat PBs.

We also showed that gliadins form dense liquid phases via the universal mechanism of liquid-liquid phase separation without the need of any ER factors. This holds true for gamma-gliadins dispersed in pure water upon micro-evaporation^72^. Above a saturation concentration, gliadins phase separate into a continuous dilute phase and dense liquid-like droplets. Thus, it is tempting to speculate that gliadins undergo liquid-liquid phase separation in ER lumen when local concentration of gliadins increases. The resulting dense liquid-like droplets may act as precursor of PBs as illustrated in Fig. 6. The liquid-like state of dense droplet would allow fast exchange and diffusion within ER lumen as observed in PBs formed in tobacco cells^9,10^. Investigating model polypeptides would help to decipher the driving force of gliadin phase separation and to identify the role of each domain on the assembly of gliadins. Indeed, growing evidences suggest that low complexity sequences, like the N-terminal domain of gliadins, are involved in liquid-liquid phase separation^12^. Also, both C-terminal and N-terminal domains have been found to induce PBs formation in tobacco leaves: they are co-localized in PBs, suggesting their ability to interact with each other^10^. Given biological complexity, further studies with increasing complexity are now needed, as it is still unclear if the assembly of wheat storage proteins into PBs is spontaneous or if it requires specific assistance from ER factors. In particular, two molecular chaperones, protein disulfide isomerase (PDI) and the binding protein (BiP), were detected in wheat endosperm. They are supposed to provide the native state of gliadins^6,73,74^ needed for the formation of protein bodies. It is however unclear if their co-localisation with PBs reflect non-specific entrapment^75^. In addition, ER membrane has also been suggested to act as an anchor for protein assembly through specific interaction with N-terminal domain of gliadins^76^.

## Conclusion

We investigated the kinetics of phase separation of wheat gliadins using TR-SALS, phase contrast microscopy and rheology. We have shown that two main patterns were observed corresponding to two mechanisms: nucleation-growth and spinodal decomposition. The investigation of the early stage of spinodal decomposition highlighted that gliadins interaction potential is more sensitive to temperature than proteins like lysozyme. It suggests an anisotropic potential with a small number of strongly attractive patches. We also showed that gliadins do not percolate in the range of (*c*, *T*) investigated despite their attractive properties. We formulate the hypothesis that repulsive clusters together with polydispersity, protein flexibility and/or hydration prevent percolation by promoting local dynamics and providing a macroscopic liquid-like behavior.

## Electronic supplementary material

Supplementary information

## Data Availability

The authors declare that all data supporting the findings of this study are available within the article and Supplementary Information, or are available from corresponding authors upon request.
