# Supplementary material for: Dynamics of liquid-liquid phase separation of wheat gliadins
Source: Sci Rep. 2018 Sep 27;8:14441. doi: 10.1038/s41598-018-32278-5 (PMC6160421; doi:10.1038/s41598-018-32278-5)
Supplement: Supplementary file 1 — Supplementary information [file 41598_2018_32278_MOESM1_ESM.docx]

**Dynamics of liquid-liquid phase separation of wheat gliadins - Supplementary Informations**

Adeline Boire, Christian Sanchez, Marie-Hélène Morel, Minne Paul Lettinga and Paul Menut

**Supplementary information (SI1)**

Temperature and concentration dependence of hydrodynamic radius of gliadins and gliadins clusters.

The objective of this supplementary information is to evaluate the effect of temperature and concentration on sizes of the two particle populations. To do so, we used dynamic light scattering as described in the Material & Method section of the paper.

1. Concentration dependence of gliadin dispersions correlogram.

Autocorrelation functions, displayed in Figure S1.1, suggest two populations of scatters in gliadin dispersions whatever the protein concentration. A Contin analysis of the experimental correlogram gives a major population with R_h,app_ = 4.3 +/-1.6 nm and a minor population with R_h_ = 99 +/- 28 nm corresponding to protein clusters. The size of these clusters does not depend on protein concentration in the range tested. It is however noticeable that the intercept of the correlogram increases as protein concentration increases. This effect is presumably induced by a better signal/noise ratio as protein concentration is increased. It is not possible to determine precisely the volume occupancy of each populations as it depends on the refraction index that we do not know for cluster phase.


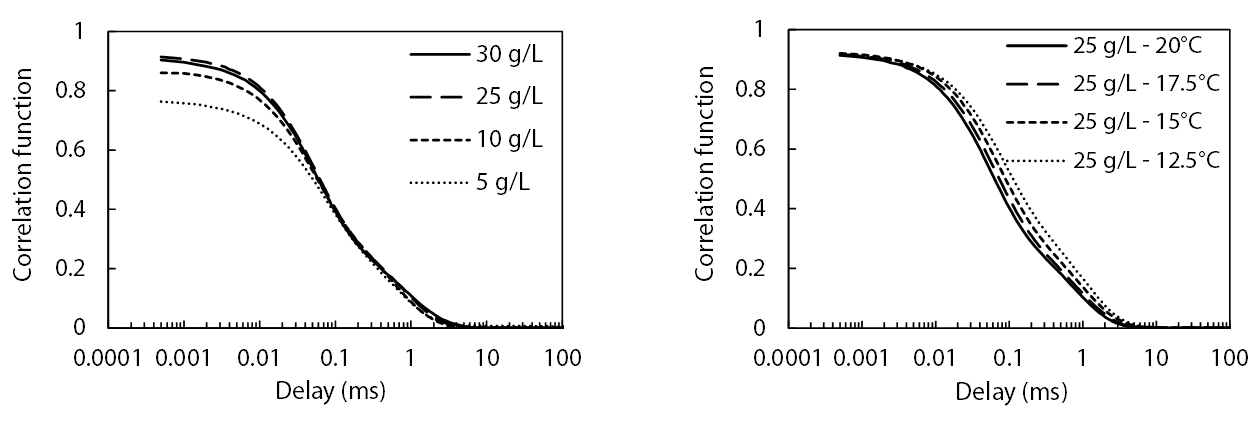


**Figure S1.1. Left: Correlation function obtained by DLS on gliadin dispersions in 55% v/v water−ethanol mixture, 0.5 mM NaCl at 20 °C at 30, 25, 10 and 5 g/L.** **Right: Correlation function obtained by DLS on gliadin dispersions at 25 g/L in 55% v/v water−ethanol mixture, 0.5 mM NaCl at 20, 17.5, 15 and 12.5°C.**

1. Temperature dependence of gliadin correlation function.

The effect of temperature on the correlation function of gliadin dispersions at 25 g/L is reported on the right panel of Figure S1.1. There is no significant change in size of the two particle populations based on a Contin analysis. The apparent shift of correlation functions towards larger delays is only due to temperature-induced viscosity effect. The contribution of clusters to the scattered intensity increases as temperature is decreased. We estimated this contribution using a simple double exponential fit as displayed on Figure S1.2.A. In this way, we obtained the temperature effect on the amplitude of each population. Results are variable from one sample to another but a tendency can be drawn: the contribution of the cluster population increases as temperature is decreased as shown in Figure S1.2.B. This could suggest an increase in the volume fraction of this population. This observation deserves further investigation.


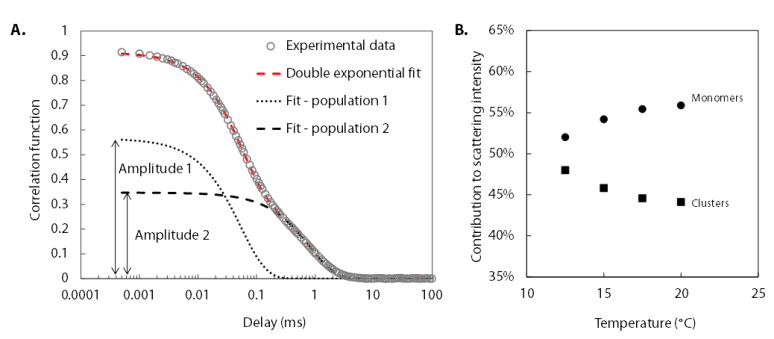


**Figure S1.2. A. Simple double exponential fit of correlation function of gliadin dispersions. B. Contribution of the two particle populations to the scattered intensity for c = 25 g/L.**

Conclusion

There is no effect of concentration and of temperature on the size of the two populations in the range tested. We will therefore use the corresponding diffusion coefficient measured by DLS. We observe however that a step-by-step temperature decrease induces an increase in the proportion of the cluster phase.

Article: Boire, A., C. Sanchez, M.-H. Morel, M. P. Lettinga and P. Menut. Dynamics of liquid-liquid phase separation of wheat gliadins.

**Supplementary information 2 (SI2)**

Determination of the effective radius of gliadins using osmotic compression experiments

We recently showed that the equation of state of gliadins (Π, Φ) deviates in several aspects to the one predicted for hard sphere dispersion^[[1]](#footnote-1)^. We highlighted, in dilute conditions for Φ < 0.04, that gliadins behave like repulsive colloids, with a positive second virial coefficient. We aim, here, to estimate the effective radius, *i.e.* the radius of the equivalent “hard-sphere” that would take into account this repulsion. To do so, we convert the osmotic pressure data into a volume fraction of equivalent hard spheres $\Phi_{eq}$ using Carnahan-Starling equation (S2.1):

$\frac{\prod}{nRT}=\frac{1+\Phi_{eq}+{\Phi_{eq}}^{2}+{\Phi_{eq}}^{3}}{\left( 1-\Phi_{eq} \right)^{3}}$ (S2.1)

The volume fraction$\Phi_{eq}$ is then converted into an equivalent radius $r_{eq}$using eq. (S2.2):

$r_{eq}=\left( \frac{\Phi_{eq}}{\frac{c}{M}\times N_{a}}\frac{3}{4\pi} \right)^{\frac{1}{3}}$ (S2.2)

Where *c* is the mass concentration, *M* the molecular weight, *N_a_* the Avogadro number. The equivalent radius depends on the volume fraction as reported on the right panel of Figure S2.1. In dilute conditions for φ < 0.04, it equals 3.3 nm +/- 0.3. It decreases down to 2.0 nm for φ = 0.2. Our data suggest an increase above φ > 0.2, but their relevance is questionable here as strong intermolecular interactions are revealed by FTIR^1^.


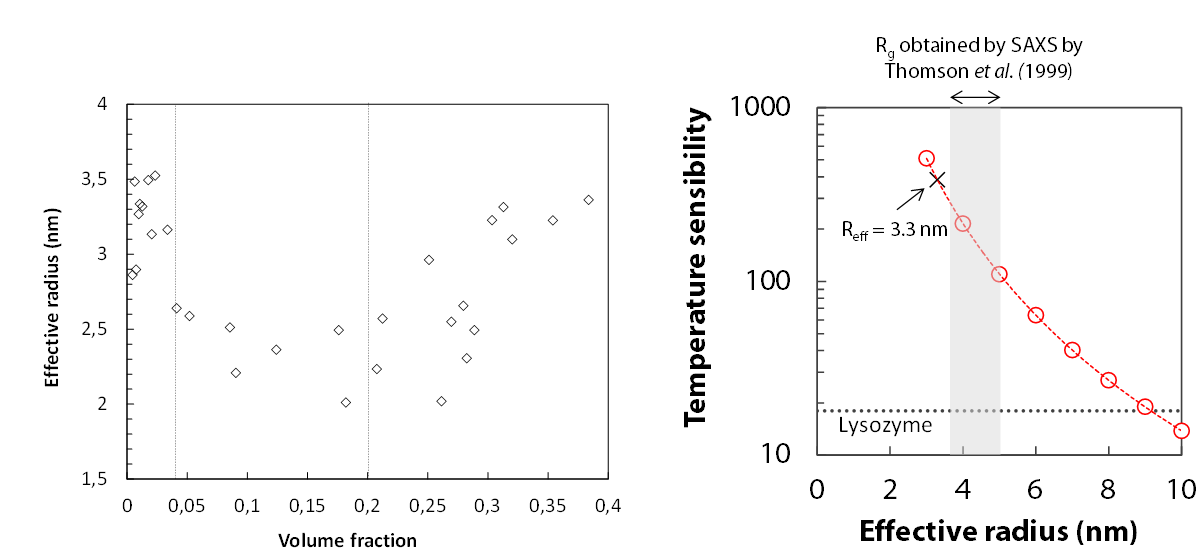


**Figure S2.1.**Right panel: Volume fraction dependence of the effective radius of gliadins. Left panel: Dependence of the temperature sensibility of gliadins B_2_* to the effective radius chosen.

The effective radius is used to calculate $B_{2,HS}$, the second virial coefficient of the equivalent hard sphere. A reduced second virial coefficient $B_{2}^{*}=B_{2}/B_{2,HS}$ is then calculated. We show in the present paper that the temperature dependence of $B_{2}^{*}$ of gliadins is much larger than the one obtained for lysozyme. The slope of B_2_*= f(T/Tc), here defined as temperature sensibility, is strongly dependent on the effective radius value, as shown on the left panel of Figure S2.1. However, we point here that (i) osmotic compression should provide a good estimate of the effective radius as it measures colligative properties, and (ii) the difference between the temperature sensibility of lysozyme and gliadins is such that even if we retain a larger radius value for gliadins, such as the one determined by Thomson *et al.* (1999)^[[2]](#footnote-2)^, the difference is still very important.

1. Boire A, Menut P, Morel M-H, Sanchez C (2015) Osmotic compression of anisotropic proteins: interaction properties and associated structures in wheat gliadin dispersions. *J Phys Chem B* 119(17):5412–5421. [↑](#footnote-ref-1)
2. Thomson, N.H., M.J. Miles, Y. Popineau, J. Harries, P. Shewry, and A.S. Tatham. 1999. Small angle X-ray scattering of wheat seed-storage proteins: alpha-, gamma- and omega-gliadins and the high molecular weight (HMW) subunits of glutenin. Biochim. Biophys. Acta. 1430: 359–366. [↑](#footnote-ref-2)
